# Feasibility of CT-derived myocardial strain measurement in patients with advanced cardiac valve disease

**DOI:** 10.1038/s41598-021-88294-5

**Published:** 2021-04-22

**Authors:** Marius Vach, Johanna Vogelhuber, Marcel Weber, Alois M. Sprinkart, Claus C. Pieper, Wolfgang Block, Daniel Kuetting, Ulrike I. Attenberger, Julian A. Luetkens

**Affiliations:** 1 Department of Diagnostic and Interventional Radiology, Quantitative Imaging Lab Bonn (QILaB), University Hospital Bonn, Venusberg-Campus 1, 53127 Bonn, Germany; 2 Department of Cardiology, University Hospital Bonn, Venusberg-Campus 1, 53127 Bonn, Germany

**Keywords:** Cardiology, Medical research

## Abstract

To explore the feasibility of CT-derived myocardial strain measurement in patients with advanced cardiac valve disease and to compare it to strain measurements derived from transthoracic echocardiography (TTE). 43 consecutive patients with advanced cardiac valve disease and clinically indicated retrospectively gated cardiac CTs were retrospectively analyzed. The longitudinal, circumferential as well as radial systolic strain were determined in all patients utilizing a commercially available CT strain software. In 36/43 (84%) patients, CT-derived longitudinal strain was compared to speckle-tracking TTE. Pearson’s correlation coefficients as well as Bland–Altman analysis were used to compare the CT-derived strain measurements to TTE. The intra- and inter-reader-reliability of the CT-derived strain measurements were assessed by intra-class correlation coefficients (ICCs). Strain measurements were feasible in all patients. CT-derived global longitudinal strain (GLS) correlated moderately with TTE-derived GLS (*r* = 0.6, *p* < 0.001). A moderate correlation between CT-derived GLS and CT-derived left ventricular ejection fraction was found (LVEF, *r* = − 0.66, *p* = 0.036). Bland–Altman analysis showed a systematic underestimation of myocardial strain by cardiac CT compared to TTE (mean difference: − 5.8%, 95% limit of agreement between − 13.3 and 1.8%). Strain measurements showed an excellent intra- and inter-reader-reliability with an intra-reader ICC of 1.0 and an inter-reader ICC of 0.99 for GLS measurements. CT-derived myocardial strain measurements are feasible in patients with advanced cardiac valve disease. They are highly reproducible and correlate with established parameters of strain measurements. Our results encourage the implementation of CT-derived strain measurement into clinical routine.

## Introduction

Myocardial strain describes the myocardial deformation throughout the heart cycle and is considered a sensitive marker for heart function. Compared to standard measurements of heart function like left ventricular ejection fraction (LVEF), the assessment of myocardial dysfunction using myocardial strain analysis adds additional diagnostic and prognostic information, which might be beneficial, especially in patients with a hypertrophic myocardium or other cardiomyopathies^1^. Global longitudinal strain (GLS) has a higher prognostic value in predicting major adverse cardiac events (MACE) than LVEF^2^. In patients under chemotherapy a decrease of GLS precedes significant changes of LVEF and helps to predict cardiotoxicity^3^. Furthermore, myocardial strain brings additional value in asymptomatic heart valve patients and helps with timing of surgery^1,4^.

The tools for measuring myocardial strain have evolved over the last decades. It all started in the late 1990s with myocardial strain measurement using Tissue Doppler echocardiography^5^. Next, the speckle-tracking algorithm was invented, which negates many disadvantages of the Tissue Doppler method and is still used today in clinical practice^1^. Nonetheless, strain measurement in echocardiography poses several problems: It is examiner dependent, not easily reproducible and depends on good image quality^6–8^.

Magnetic resonance imaging (MRI) is another imaging modality, which allows myocardial strain measurements and negates some disadvantages of echocardiography. There are many techniques for measuring strain in cardiac MRI that use specific pulse sequences^9^. To obtain strain measurements from routine cine sequences the feature-tracking algorithm was applied to cardiac cine sequences^10^. This algorithm is similar to the speckle-tracking algorithm and tracks small “features” of the myocardium, e.g. small voxel patterns, to measure the deformation of the myocardium throughout the heart cycle.

Recent software developments also allow the assessment of myocardial strain from cardiac computed tomography (CT)^11^. While there are some studies that show that myocardial strain imaging in CT using the feature-tracking approach is possible, data on CT-based strain measurements is still limited and has been validated primarily in patients with severe aortic stenosis^11–14^.

The aim of this study is to evaluate whether measuring myocardial strain from retrospectively gated CT scans using the feature-tracking method with commercially available software is feasible in patients with advanced cardiac valve disease. Furthermore, the CT-derived myocardial strain is compared to speckle-tracking based echocardiography.

## Methods

### Study population

This retrospective study was approved by the institutional review board of the University Bonn with waiver of written informed consent and hence all methods were performed in compliance with the ethical standards set in the 1964 Declaration of Helsinki as well as its later amendments. The study population consisted of 43 consecutive patients who received a clinically indicated cardiac CT between 06/2018 and 02/2019 at our institution for planning an intervention of the mitral or tricuspid valve as well as evaluation of the aortic valve. CT examinations were screened for appropriate contrast as well as complete coverage of the left ventricle by a radiologist with two years of experience in cardiac CT. Then medical records were reviewed, and baseline characteristics were obtained.

### 2D echocardiography

Two-dimensional ECG-gated transthoracic echocardiography (TTE) studies were performed with commercially available ultrasound systems (Epiq CVX, Philips Medical Systems, Andover, Massachusetts and Vivid E95, GE Healthcare Vingmed Ultrasound, Horten, Norway) with standard transducers for echocardiography (> 30 Hz resolution). Three consecutive cardiac cycles were recorded in three apical views (two-, three- and four-chamber views). For assessment of longitudinal strain, recordings were processed with a dedicated, commercially available software for strain analysis (TomTec Imaging Systems GmbH, Unterschleissheim, Germany) allowing offline semi-automatic myocardial strain assessment using the speckle-tracking method. The endocardial border of the end-diastolic left ventricle was manually traced using point-and-click technique and visually controlled and revised, if necessary. Left ventricular ejection fraction was quantified using the modified Simpson method.

### CT imaging protocol and image reconstruction

Retrospectively ECG-gated 192-slice cardiac CT examinations were performed with a dual-source CT scanner (Somatom Force, Siemens Medical Solutions, Forchheim, Germany). The tube voltage was 100 kV and the tube current was modulated automatically. Detector collimation was 192 × 0.6 mm.

80–100 ml of iodinated contrast medium (Ultravist 300, Bayer Vital, Leverkusen, Germany) was applied with either a triphasic or quadruple-phase injection protocol, depending on the indication. The triphasic injection protocol consisted of 80 ml of contrast medium (CM) followed by 60 ml of 30%/70% diluted CM/saline and finally a saline chaser bolus of 30 ml. The quadruple-phasic injection protocol was used in patients with tricuspid regurgitation to optimize the contrast in the right ventricle. 40 ml of CM was followed by 60 ml of 30%/70% diluted CM/saline with another 20 ml of pure CM and a 30 ml saline chaser bolus after that. The flow was between 5 and 6 ml/s, depending on the body mass index (BMI) of the patient, in all cases. To time the scan Bolus-tracking in the descending aorta with a threshold of 150 Hounsfield units (HU) and a threshold delay of 10 s was used.

Image series were reconstructed in 5% steps throughout the heart cycle resulting in 20 images per heart cycle. Two-, three- and four-chamber cine views were reconstructed with a dedicated post-processing software (Intellispace Portal, Philips Healthcare, Best, The Netherlands) and exported as DICOM files. Short-axis views were reconstructed from transversal images in the analysis software (Segment CT, Medviso, Lund, Sweden). The left ventricle in the short axis view was segmented semi-automatically by the software and corrected manually if necessary^15^.

### Evaluation of two-dimensional strain with feature tracking algorithm with 2D-CCT

The used feature-tracking software (Segment CT, Medviso, Lund, Sweden) is commercially available and CE-certified. It allows measurement of longitudinal, circumferential and radial strain as well as the strain rate in short-axis and long-axis cine views.

After importing the images as DICOM files, the myocardium of the left ventricle was outlined in end-diastolic short-axis as well as long-axis cine views by a radiologist with two years of experience in cardiac CT. The strain was then calculated inside the delineated myocardium.

The algorithm is based on an elastic image registration approach and tracks so-called features of the heart, e.g. the endocardial border, papillary muscles or trabeculae, and traces their movement through the heart cycle. This allows the calculation of the strain in three different orientations (longitudinal, radial and circumferential). The software tool estimates the myocardial strain by computing inter-frame deformation maps using a tracking strategy based on elastic image registration^16^. The feature tracking algorithm was initially developed for cardiac MRI and recently adopted for cardiac CT. The details of the algorithm have been described before and it has been validated before in cardiac MRI^16,17^.

The strain assessment was done in three short-axis slices (basal, midventricular and apical) as well as in the two-, three- and four-chamber long-axis views. All these views are standardized and therefore allow a comparison of strain parameters between cardiac CT and echocardiography. For segmental analysis the myocardium was divided in 17 segments according to the model of the American Heart Association. The software reports a strain value for every segment. These values of all segments were then used to calculate the global peak strain in longitudinal, radial and circumferential orientation. The global strain represents the mean of all peak strains of every segment in the respective orientation. We analyzed these “global” strain measurements of the left ventricle.

### Statistical analysis

Statistical analysis was performed with the Software Package R and a specific plotting library (ggplot2 package, Hadley Wickham, 2016, New York, NY, USA and R Foundation for Statistical Computing, 2018, Vienna, Austria). Continuous variables are presented as mean ± standard deviation. Continuous data that deviates significantly from a normal distribution are presented as median and range. Normal distribution was assessed using the Shapiro–Wilk test. Categorical data are given as frequencies. Pearson’s correlation coefficient was obtained to compare the different strain measurements in CT and TTE as well as to compare the CT-derived strain measurements with the CT- and TTE-derived left ventricular ejection fraction (LVEF). Additionally, a Bland–Altman analysis was conducted to compare the myocardial strain measurements in cardiac CT and TTE. A *p*-value of < 0.05 was considered statistically significant.

A random subset of 10 patients was chosen to test the intra- and inter-reader-repeatability of the CT-derived strain measurements. Intra-reader repeatability was tested by one reviewer (MV) by analyzing the subset of 10 patients again several weeks after the initial analysis. For inter-reader repeatability another reviewer (DK) analyzed the images of the subset of 10 patients. The repeatability was assessed by a two-way random intra-class correlation coefficient (ICC).

## Results

### Patient characteristics and radiation dose

**Figure 1:**
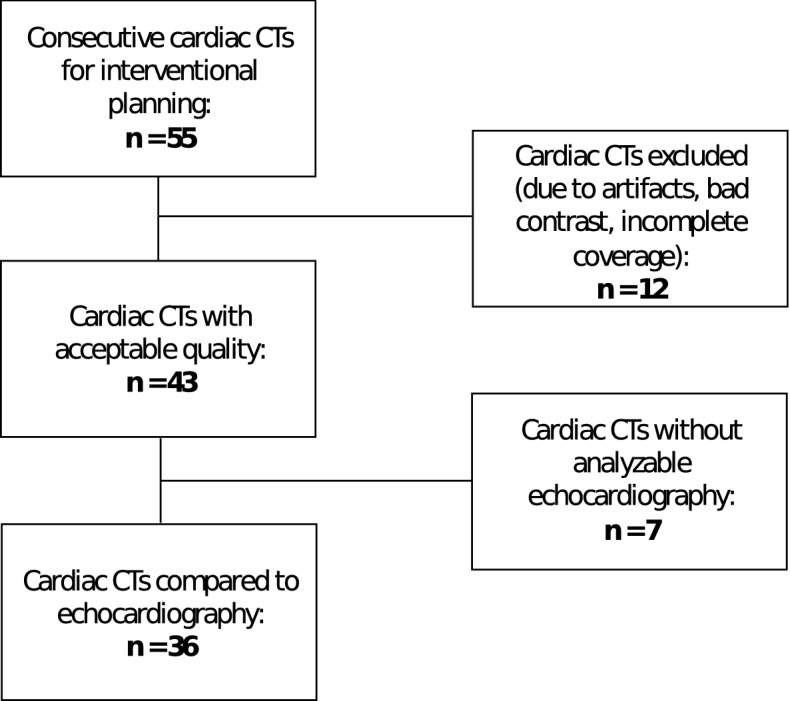
Flow chart of the patients in the study. 55 consecutive cardiac CTs, which were obtained for interventional planning of the cardiac valves were screened and 12 CTs were excluded due to either severe artifacts, bad contrast or incomplete coverage of the left ventricle. In the remaining 43 cardiac CTs myocardial strain was assessed. 7 of these 43 cardiac CTs had no analyzable echocardiography. In the remaining 36 examinations the myocardial strain measurements between echocardiography and cardiac CT were compared.

In total 43 patients were included in this retrospective analysis (see Fig. 1). 17 patients were male (40%). The indication for the cardiac CT was evaluation of mitral regurgitation in 34 (79%) cases, evaluation of tricuspid regurgitation in 6 (14%) cases and other reasons in 3 (7%) cases. The other reasons were evaluation before transcatheter aortic valve replacement (TAVR), valve thrombosis after TAVR and fibroelastoma of the aortic valve. 26/43 (60%) patients were female. Mean age was 71.0 ± 13.6 years with a range between 39 and 95 years. BMI was between 20.8 and 34.1 kg/cm^2^ (mean: 27.8 ± 4.6 kg/cm^2^). Mean dose length product (DLP) for cardiac CT was 1021.2 ± 561.9 mGy*cm. The median time between cardiac CT and TTE was 8 days (range 0–156 days). No changes of medication or cardiovascular events occurred between the cardiac CT and echocardiography examinations. Clinical characteristics of the patients are given in Table 1.

**Table 1:** Demographic and baseline characteristics.

Patients	n = 43
Age (years)	70.95 ± 13.58
Male [n (%)]	17 (39.5)
BMI (kg/m^2^)	27.76 ± 4.55
DLP (mGy*cm)	1021.24 ± 561.92
Days between TTE and CT [median (range)]	8 (0–156)
Chronic kidney disease [n (%)]	10 (25.0)
DM [n (%)]	13 (32.5)
AF [n (%)]	27 (65.9)
Hypertension [n (%)]	28 (68.3)
Dyslipidemia [n (%)]	15 (36.6)
Prior MI [n (%)]	5 (12.2)
COPD [n (%)]	4 (9.8)
TAVR [n (%)]	4 (9.3)
PM/ICD [n (%)]	8 (20.0)
Creatinine (mg/dl)	1.51 ± 1.34
CRP (mg/l)	11.17 ± 12.26
NTproBNP (pg/ml)	2773.50 ± 3192.38
**Cardiac medication**	
Diuretics [n (%)]	36 (87.8)
ACE inhibitors [n (%)]	12 (29.3)
Beta blockers [n (%)]	32 (80.0)
Anticoagulants [n (%)]	30 (73.2)

*BMI* body mass index, *DLP* dose length product, *DM* diabetes mellitus, *AF* atrial fibrillation, *MI* myocardial infarction, *COPD* chronic obstructive pulmonary disease, *TAVR* transcatheter aortic valve replacement, *PM* pacemaker, *ICD* Implantable cardioverter-defibrillator, *CRP* C-reactive protein, *NTproBNP* B-type natriuretic peptide.

### Relation of CT-derived strain and LVEF

**Figure 2:**
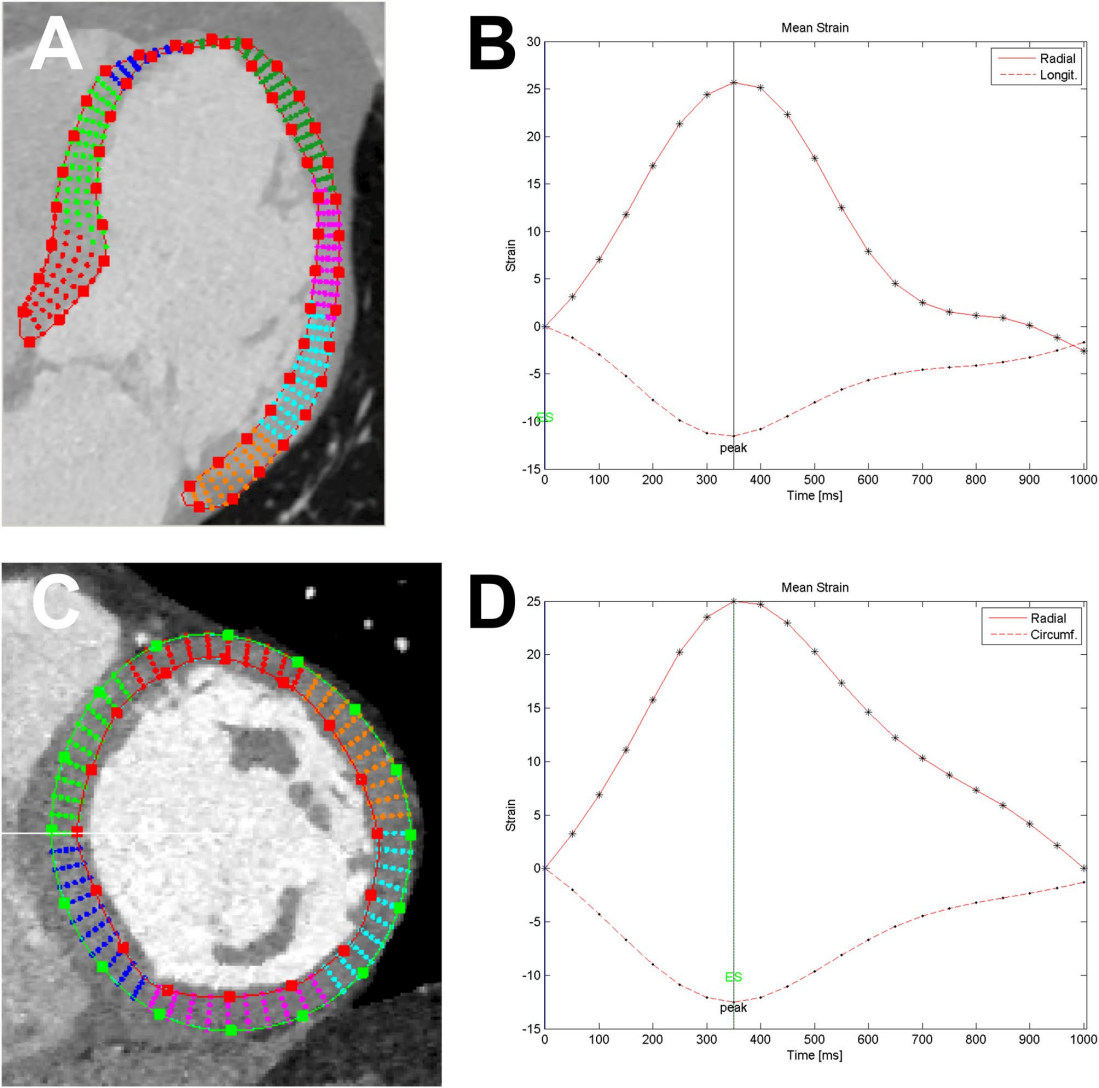
Example of strain measurement in SegmentCT (Medviso, Lund, Sweden). Images (**A**) and (**B**) show an example of strain measurement in a four-chamber view of the left ventricle with the corresponding strain curve for radial and longitudinal strain. Images (**C**) and (**D**) show an example of strain measurements in a short axis view of the left ventricle with the corresponding strain curves for radial and circumferential strain.

**Figure 3:**
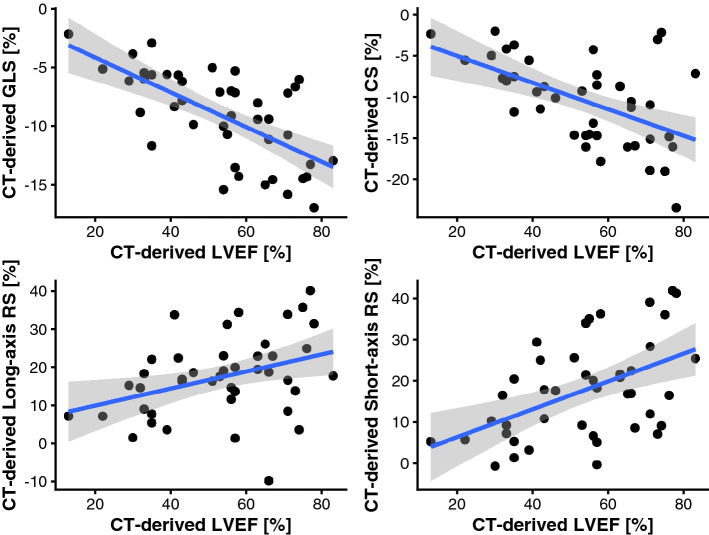
Scatterplot showing the correlation of CT-derived strain in different orientations and CT-derived LVEF. Pearson’s correlation coefficients for the different orientations are as follows: GLS: *r* = − 0.66 (*p* < 0.001), CS: *r* = − 0.53 (*p* < 0.001), long-axis RS: *r* = 0.37 (*p* = 0.02), short-axis RS: *r* = 0.49 (*p* < 0.001; GLS, global longitudinal strain; CS, circumferential strain; RS, radial strain; LVEF, left ventricular ejection fraction).

An example of the strain measurements in Segment CT (Medviso, Lund, Sweden) can be found in Fig. 2. There was a moderate correlation between CT-derived GLS and CT-derived LVEF (*r* = − 0.66, *p* < 0.001). Correlation parameters for the individual long axis views are provided in Table 2. Circumferential strain correlated moderately with the CT-derived LVEF (*r* = − 0.53, *p* < 0.001). Similar results were observed for radial strain derived from short axis view (*r* = 0.49, *p* < 0.01) as well as derived from the long-axis (*r* = 0.37, *p* = 0.02). A scatterplot with CT-derived myocardial strain measurements and the CT-derived LVEF can be found in Fig. 3.

**Table 2:** Correlation between CT-derived longitudinal strain and CT-derived LVEF in different orientations as well as globally.

Orientation	Pearson’s *r* for CT-LS versus CT-LVEF
Global	− 0.66
Two-chamber view	− 0.63
Three-chamber view	− 0.64
Four-chamber view	− 0.60

*LS* longitudinal Strain, *LVEF* left ventricular ejection fraction.

### Relation of CT-derived and TTE-derived longitudinal strain

**Figure 4:**
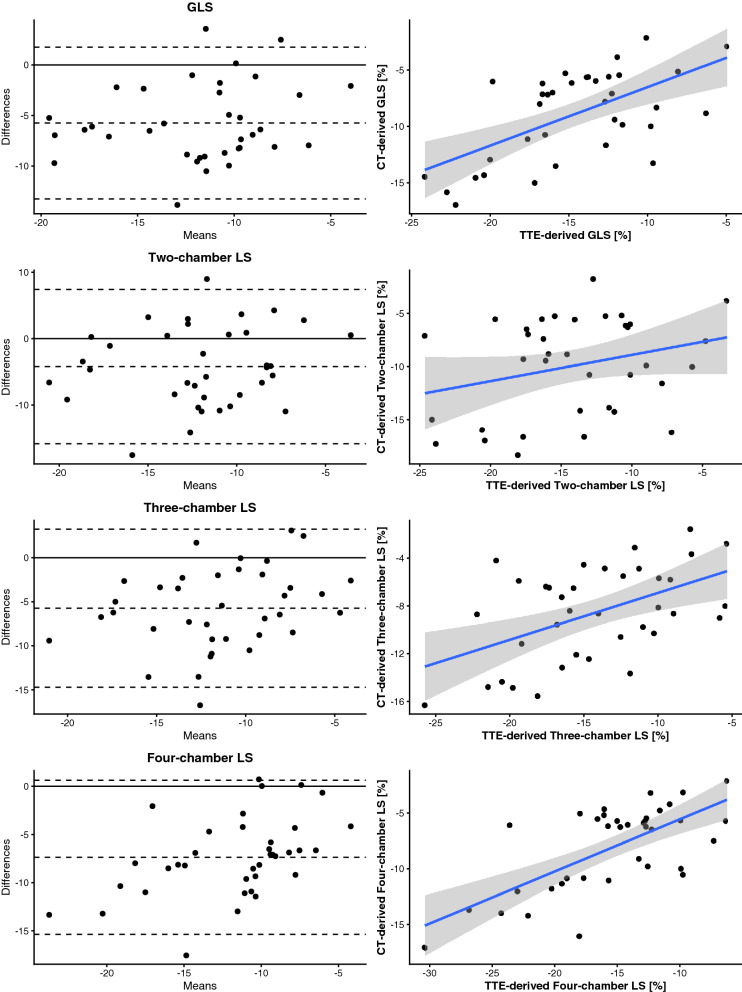
Scatterplots and Bland–Altman plots of global longitudinal strain as well as longitudinal strain in the two-, three- and four-chamber view. The Pearson’s correlation coefficient was as follows: global longitudinal strain: *r* = 0.60, *p* < 0.001; two-chamber view: *r* = 0.28, *p* = 0.0929; three-chamber view: *r* = 0.51, *p* = 0.0014; four-chamber view: *r* = 0.69, *p* < 0.001. (GLS, global longitudinal strain; LS, longitudinal strain; TTE, trans-thoracic echocardiography).

36 patients had both, an evaluable CT and an evaluable echocardiogram. Mean CT-derived GLS was − 9.1 ± 3.9% while the mean TTE-derived GLS was − 14.7 ± 4.6%. Mean CT-derived LVEF was 52.8 ± 16.6% and the mean TTE-derived LVEF was 51.4 ± 11.9%. CT-derived LVEF and TTE-derived LVEF correlated strongly (*r* = 0.75, *p* = 0.012). There was a moderate correlation between TTE-derived GLS and CT-derived GLS (*r* = 0.60, *p* < 0.001). The correlation differed strongly when comparing the longitudinal strain (LS) in the different long axis views (two-chamber view: *r* = 0.28, *p* = 0.093; three-chamber view: *r* = 0.51, *p* = 0.0014; four-chamber view: *r* = 0.69, *p* < 0.001; see Table 3). Bland–Altman analysis showed a systematic underestimation of LS in cardiac CT (mean difference for GLS is − 5.8% with a 95% limit of agreement between − 13.3 and 1.8%). A scatterplot with the linear best-fit line as well as a Bland–Altman plot for the different long axis views as well as the GLS can be seen in Fig. 4. The CT-derived GLS correlated moderately with the TTE-derived LVEF (*r* = − 0.53, *p* < 0.001).

**Table 3:** Pearson’s correlation coefficient between CT-derived and TTE-derived longitudinal strain.

Orientation	Pearson’s *r* for CT-LS versus TTE-LS
Global	0.60
Two-chamber view	0.28
Three-chamber view	0.51
Four-chamber view	0.69

*LS* longitudinal strain.

### Intra- and inter-rater reproducibility

The strain measurements showed an excellent intra- and inter-reader reproducibility. The intra-class correlations for every strain measurement are provided in Table 4.

**Table 4:** Intra-class correlation coefficients (ICC) for intra-reader as well as inter-reader reliability of the different CT-derived strain measurements.

Parameter	Intra-reader ICC	Inter-reader ICC
Global LS	1.00	0.99
Four-chamber LS	1.00	1.00
Three-chamber LS	1.00	0.99
Two-chamber LS	0.97	0.96
Circumferential strain	0.99	0.99
Short-axis radial strain	0.99	0.99
Long-axis radial strain	0.78	0.78

*LS* longitudinal strain.

## Discussion

This is the first study to assess the viability of measuring myocardial strain in cardiac CT in patients with advanced cardiac valve disease and compare it to TTE. Our major findings are:

- Myocardial strain measurement in cardiac CT is feasible and CT-derived myocardial strain correlates moderately with CT-LVEF as well as TTE-derived myocardial strain and LVEF, but a variability exists between the two modalities
- CT underestimates strain compared to TTE
- Measuring strain in CT shows a high intra- and inter-reader-reliability

Myocardial strain has prognostic value in patients with advanced cardiac valve disease, e.g. severe mitral regurgitation. GLS predicts a reduction of LVEF after mitral valve replacement as well as the post-procedural outcome^18,19^. More and more patients with severe cardiac valve disease are treated interventionally. Since these patients receive a cardiac CT for interventional planning as part of their routine diagnostic work-up and myocardial strain has been shown to be a good outcome prediction parameter in these patients, it is of utmost interest to evaluate the feasibility of “opportunistic” myocardial strain assessment from these planning CTs. This could lead to a better patient selection for these interventions.

However, the feasibility of CT-based myocardial strain assessment in patients with severe tricuspid or mitral regurgitation has not been studied yet.

The feasibility of myocardial strain measurement in cardiac CT has been studied mainly in patients with severe aortic stenosis^11,14,20,21^. The largest study to date examined 123 patients with severe aortic stenosis and compared the strain assessment in cardiac CT and TTE before TAVR^21^. The authors found a greater GLS in CT than in TTE with a moderate correlation between the two modalities (*r* = 0.62). There was a strong correlation between GLS and LVEF within one modality (*r* = − 0.9 for CT and *r* = − 0.88 for TTE). The correlation of GLS between the two modalities is comparable to our results, although we only found a moderate correlation between CT-derived GLS and LVEF. In general, the correlation coefficients for CT- and TTE-derived GLS reported in the literature range from 0.62 to 0.84^11–14,21,22^. Since the correlation coefficient in our analysis is comparable to the literature, we conclude that the software tool we used is at least comparable to other CT strain tools. This moderate correlation between CT-derived myocardial strain and TTE-derived myocardial strain shows that those two modalities are not merely interchangeable and that there is a significant variability^21^. A comparison of cardiac MRI (CMR) based feature-tracking with the same software tool used in this study and echocardiography showed a comparable correlation coefficient of *r* = 0.74^23^.

Although speckle-tracking and feature-tracking are similar approaches to assessing the myocardial deformation through the heart cycle, there are inherent differences between the two approaches. While speckle-tracking tracks intramyocardial patterns throughout the heart cycle, which is also possible with feature-tracking algorithms, most feature-tracking algorithms follow recognizable cardiac structures like the endocardial border through the heart cycle. This lack of intramyocardial features explains why some differences between the two modalities are to be expected 8. However, technically, it is important to mention in this context that although feature tracking often tracks a peculiar pattern along a curve of an image (e.g. papillary muscles or endocardial boarders), image features are characteristics of the intensities in a localized region (mean intensity, intensity standard deviations, Law’s texture energy, etc.). The lack of intramyocardial features explains why some differences between the two modalities are to be expected^8^. But even when comparing the feature-tracking method between cardiac MRI and CT there is only a moderate correlation between the two modalities^20^. This further proves the point that, although measuring the same phenomenon, myocardial strain values between different modalities cannot easily be compared.

The correlation of the longitudinal strain measurements between cardiac CT and echocardiography varied considerably depending on which long-axis view was compared and was the highest when comparing the four-chamber views of cardiac CT and echocardiography. Possible reasons might be a better anatomical depiction and reproducibility of the four-chamber view in echocardiography with more altering image quality of the other long-axis views.

This is the first work to use this specific software tool (Segment CT, Medviso, Lund, Sweden) for myocardial strain assessment in cardiac CT. Since every software tool uses its own algorithm, which might track the myocardium a little bit differently, different software tools may produce slightly different strain values, although fundamentally measuring the same phenomenon as has been reported for echocardiography before^24,25^.

We observed a systematic underestimation of myocardial strain by CT compared to TTE. This has been reported before and might be explained by the lower temporal resolution of CT compared to TTE^8,22^. Although the temporal resolution in our case with 20 images per heart cycle was higher than in previous studies, there is still a difference compared to TTE. This is another reason why CT-derived strain should be independently validated and the normal values from one modality cannot be transferred to another.

Nonetheless, the strain measurements in CT are highly reliable between different readers as well as when comparing multiple measurements of the same reader.

Cardiac CT is the only diagnostic tool that can simultaneously image the general heart anatomy, the coronary arteries, the myocardium and assess the cardiac function of the patient. The emergence of myocardial strain further proves the trend of cardiac CT becoming a “one-stop shop” for cardiac diagnostics. A problem that remains (for now) is the relatively high radiation exposure that is needed for a retrospectively gated, full heart cycle cardiac CT, which is needed for functional analysis. The radiation exposure of the examinations in our study was around 18 mSv. Since the whole heart cycle needs to be imaged, radiation saving techniques like step-and-shoot or high pitch spiral acquisition (e.g. FLASH by Siemens) cannot be used. While the radiation dose of cardiac CT is too high to justify a CT scan for the assessment of myocardial strain alone, the authors believe that the technique of cardiac CT strain measurements should rather be “opportunistically” used in patients with a clear clinical indication for cardiac CT (e.g. patients with severe cardiac valve disease). In these patients, myocardial strain measurements might be additionally performed and could provide additional prognostic value and guide treatment decision in the future. But further technical improvements are expected to lower the radiation dose and will help to establish cardiac CT in more clinical indications.

Although cardiac CT is much more resilient in terms of image quality compared to echocardiography, especially cardiac implants like pacemaker electrodes or heart valves can cause artefacts that obscure parts of the myocardium. The effect of these implants on the myocardial strain assessment in cardiac CT is largely unknown. We believe it is crucial to assess whether cardiac implants like pacemaker electrodes have an effect on the myocardial strain assessment in cardiac CT because these are the patients that have a contraindication for cardiac MRI and are therefore candidates for functional assessment in cardiac CT.

### Limitations

This work needs to be seen in the light of some limitations: While myocardial strain is a regional marker of myocardial function, we have only examined global strain measurements, which does not allow conclusions about regional myocardial strain. However, global myocardial strain parameters of the left ventricle have been proven to be a strong outcome predictor in different disease settings^1,2^. Therefore, the assessment of global strain values might be clinically more applicable. The time between cardiac CT and TTE varied between patients and for some patients the time period between CT and TTE was slightly longer, which bares the risk of varying cardiac function between the two examinations. Nonetheless, in previous studies myocardial strain has shown an excellent test–retest reproducibility^26^. Furthermore, the cohort was rather small with 43 subjects and only 36 patients in whom cardiac CT and TTE could be compared. No comparison between cardiac CT and MRI could be obtained in the present study. This would be an interesting question for further research. Unfortunately, we were only able to assess the longitudinal strain in TTE and therefore only a comparison of this type of myocardial strain was possible between the two modalities. Lastly, we did not use the same software tool for CT and TTE, which might hinder the comparability of the strain measurements. Lastly, myocardial strain assessment in our study is based on 2D based tracking which neglects the out-of-plane element of the myocardial motion vectors. New CT-based approaches for myocardial strain assessment use the full 3D information of cardiac CT to obtain 3D based strain measurements^27,28^. Nonetheless, 2D based strain is the most validated and provides value to patients with different cardiac diseases^1,2^.

### Conclusion

CT-derived myocardial strain measurement is feasible showing a high inter- and intra-reader-repeatability and a moderate correlation with echocardiography-derived myocardial strain. CT-derived myocardial strain is a promising CT-derived parameter for heart function and should be further validated in different clinical settings.

## Data Availability

The datasets generated during and/or analyzed during the current study are available from the corresponding author on reasonable request.

## Abbreviations

CT: Computed tomography
TTE: Trans-thoracic echocardiography
LVEF: Left ventricular ejection fraction
GLS: Global longitudinal strain
LS: Longitudinal strain
MACE: Major adverse cardiac events
ECG: Electrocardiogram
MRI: Magnetic resonance tomography
CM: Contrast medium
ICC: Intra-class correlation coefficient
TAVR: Transcatheter aortic valve replacement
DLP: Dose length product
BMI: Body mass index
HU: Hounsfield units
